# Indications for medical compression stockings in venous and lymphatic disorders: An evidence-based consensus statement

**DOI:** 10.1177/0268355516689631

**Published:** 2017-02-22

**Authors:** Eberhard Rabe, Hugo Partsch, Juerg Hafner, Christopher Lattimer, Giovanni Mosti, Martino Neumann, Tomasz Urbanek, Monika Huebner, Sylvain Gaillard, Patrick Carpentier

**Affiliations:** 1 Department of Dermatology, University of Bonn, Bonn, Germany; 2 Department of Dermatology, Medical University of Vienna, Austria; 3 Department of Dermatology, University Hospital of Zurich, Zurich, Switzerland; 4 Josef Pflug Vascular Laboratory, West London Vascular and Interventional Centre, Ealing Hospital & Imperial College, London, UK; 5 Angiology Department, Clinica MD Barbantini, Lucca, Italy; 6 Department of Dermatology, Erasmus University Hospital, Rotterdam, The Netherlands; 7 Medical University of Silesia Department of General Surgery, Vascular Surgery, Angiology and Phlebology, Katowice, Poland; 8 SIGVARIS AG, St Gallen, Switzerland; 9 SIGVARIS Management AG, Winterthur, Switzerland; 10 Centre de Recherche Universitaire de La Léchère, Equipe THEMAS, Université Joseph Fourier, Grenoble, France

**Keywords:** Compression stockings, chronic venous disease, deep vein thrombosis, post-thrombotic syndrome, lymphoedema

## Abstract

**Objective:**

Medical compression stockings are a standard, non-invasive treatment option for all venous and lymphatic diseases. The aim of this consensus document is to provide up-to-date recommendations and evidence grading on the indications for treatment, based on evidence accumulated during the past decade, under the auspices of the International Compression Club.

**Methods:**

A systematic literature review was conducted and, using PRISMA guidelines, 51 relevant publications were selected for an evidence-based analysis of an initial 2407 unrefined results. Key search terms included: ‘acute', CEAP', ‘chronic', ‘compression stockings', ‘compression therapy', ‘lymph', ‘lymphatic disease', ‘vein' and ‘venous disease'. Evidence extracted from the publications was graded initially by the panel members individually and then refined at the consensus meeting.

**Results:**

Based on the current evidence, 25 recommendations for chronic and acute venous disorders were made. Of these, 24 recommendations were graded as: Grade 1A (n = 4), 1B (n = 13), 1C (n = 2), 2B (n = 4) and 2C (n = 1). The panel members found moderately robust evidence for medical compression stockings in patients with venous symptoms and prevention and treatment of venous oedema. Robust evidence was found for prevention and treatment of venous leg ulcers. Recommendations for stocking-use after great saphenous vein interventions were limited to the first post-interventional week. No randomised clinical trials are available that document a prophylactic effect of medical compression stockings on the progression of chronic venous disease (CVD). In acute deep vein thrombosis, immediate compression is recommended to reduce pain and swelling. Despite conflicting results from a recent study to prevent post-thrombotic syndrome, medical compression stockings are still recommended. In thromboprophylaxis, the role of stockings in addition to anticoagulation is limited. For the maintenance phase of lymphoedema management, compression stockings are the most important intervention.

**Conclusion:**

The beneficial value of applying compression stockings in the treatment of venous and lymphatic disease is supported by this document, with 19/25 recommendations rated as Grade 1 evidence. For recommendations rated with Grade 2 level of evidence, further studies are needed.

## Introduction

Compression therapy for the management of chronic venous disease (CVD) and lymphoedema is a readily available, non-invasive treatment option which is widely practiced and extensively documented.^1–3^ However, there is a paucity of guidelines derived from evidence reported in clinical trials for the management of patients using compression therapy.^4^ Currently, the majority of recommendations are based on expert opinion rather than evidence. The purpose of this document was to review the current evidence from the literature supporting compression performed by medical compression stockings (MCS) and use this to compile and grade recommendations for use in clinical practice.

The International Compression Club (ICC) is an ad hoc group of experts that includes clinicians involved in compression therapy, as well as technical specialists from companies manufacturing compression devices for clinical use. The ICC published a consensus statement in 2008 on the use of compression therapy in the management of venous and lymphatic diseases.^4^ The aim of this article is to provide an update of the 2008 consensus statement in order to answer the clinical questions that were outstanding. Importantly, the 2008 consensus statement was based on the clinical manifestation (C) stages of the Clinical-Etiology-Anatomy-Pathophysiology (CEAP) classification.^5^ However, this consensus statement is based on the clinical goals of treatment, with recommendations based on the primary outcome of compression treatment focusing on MCS only. The recommendations in this consensus document are not intended to establish the superiority of MCS or compression pressures over other compression devices or treatment modalities.

## Methods

### Consensus panel

The consensus group that contributed to this statement comprised eight international experts experienced in compression therapy, representing different medical disciplines (angiology, dermatology and vascular surgery). They were selected by Eberhard Rabe (the primary author) who appointed a consensus meeting Chair, Hugo Partsch, and the remaining members. The responsibility of the Chairs, supported by a secretary, Sylvain Gaillard, was to perform and refine the literature searches, to use their personal records and knowledge, and to select/compose the initial recommendations as a framework for discussion.

### Study design

**Figure 1.**
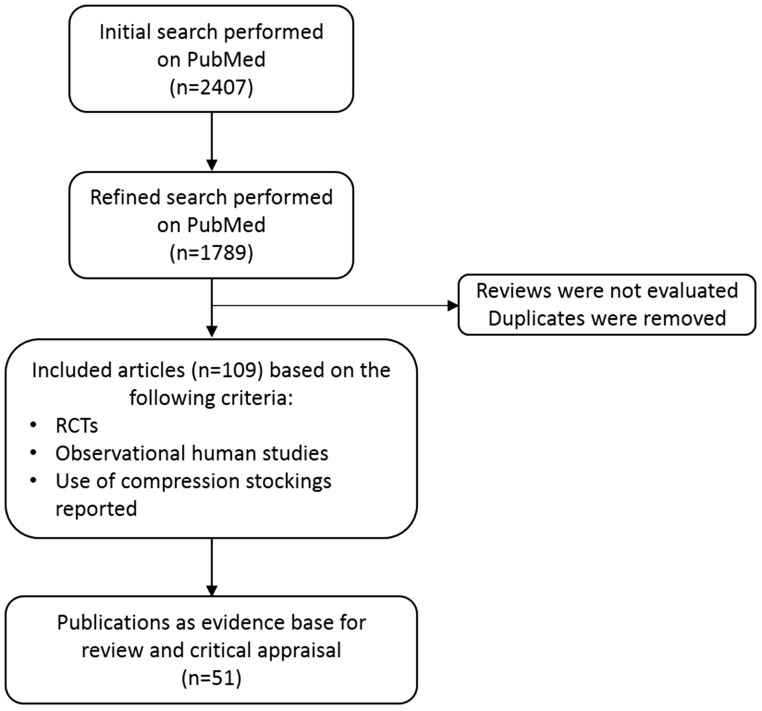
PRISMA flow diagram of relevant literature identified. PRISMA: Preferred Reporting Items for Systematic reviews and Meta-Analyses; RCTs: randomised controlled trial.

A systematic literature search was performed for articles published between 1 January 2007 and 8 July 2015 using PubMed. Key search terms included ‘acute’, ‘CEAP’, ‘chronic’, ‘compression therapy', ‘compression stockings', ‘lymph', ‘lymphatic disease', ‘vein' and ‘venous disease'. The searches produced 2407 results initially and, subsequently, a total of 1789 results, after refinement (Figure 1).

Studies were deemed eligible if they were randomised controlled trials (RCTs) or observational studies reporting on the use of MCS and published in the English language. Published evidence on the effectiveness of MCS was reviewed and critically appraised by the Chairs, who concentrated on RCTs that addressed the use of compression for a large number of clinical indications. In addition, the consensus group members searched their personal collections of papers for relevant medical literature. Reviews were not evaluated and duplicate publications within the CEAP classes were excluded. The results of this search strategy were further refined by the consensus meeting Chairs, who manually selected a total of 109 potentially relevant publications from the list, as well as from an additional meta-analysis. After a decision to include only literature on compression stockings, a further review by the consensus meeting Chairs identified 51 relevant publications, which were selected as the evidence base for review and critical appraisal.

The evidence was graded according to the recommendations of Guyatt et al.^6^ (Table 1). For this, ratings of ‘high’, ‘moderate’ or ‘low’ were given to three core components of the respective studies: study quality; risk of bias; and benefit versus risk. An initial grading of the evidence and preliminary recommendations was developed by the meeting Chairs. The task of making the final selection of papers was divided amongst all the panel members, with reference to the recommendations on grading. In September 2015, all panel members met in Frankfurt to share their opinions and reach a consensus on a set of recommendations and on their final grading of evidence, using the preliminary recommendations and initial grading. In finalising their proposals, the panel used the 2008 ICC-consensus statement as a basis and concentrated on new publications that were published since 1 January 2007.

**Table 1.** Grading recommendations.^6^

Grade	Description of recommendation	Benefit vs. risk	Methodological quality of supporting evidence	Implications
1A	Strong recommendation, high-quality evidence	Benefits clearly outweigh risk and burdens, or vice versa	RCTs without important limitations or overwhelming evidence from observational studies	Strong recommendation, can apply to most patients in most circumstances without reservation
1B	Strong recommendation, moderate-quality evidence	Benefits clearly outweigh risk and burdens, or vice versa	RCTs with important limitations (inconsistent results, methodological flaws, indirect, or imprecise) or exceptionally strong evidence from observational studies	Strong recommendation, can apply to most patients in most circumstances without reservation
1C	Strong recommendation, low-quality or very low-quality evidence	Benefits clearly outweigh risk and burdens, or vice versa	Observational studies or case series	Strong recommendation but may change when higher-quality evidence is available
2A	Weak recommendation, high-quality evidence	Benefits closely balanced with risks and burden	RCTs without important limitations or overwhelming evidence from observational studies	Weak recommendation; best action may differ depending on circumstances or patients’ or societal values
2B	Weak recommendation, moderate-quality evidence	Benefits closely balanced with risks and burden	RCTs with important limitations (inconsistent results, methodological flaws, indirect, or imprecise) or exceptionally strong evidence from observational studies	Weak recommendation; best action may differ depending on circumstances or patients’ or societal values
2C	Weak recommendation, low-quality or very low-quality evidence	Uncertainty in the estimates of benefits, risks and burden; benefits, risk and burden may be closely balanced	Observational studies or case series	Very weak recommendations; other alternatives may be equally reasonable

## Recommendations

### Patients with chronic venous disorders and healthy individuals

#### Improvement of venous symptoms, quality of life and oedema

Venous symptoms, which include heaviness and tension or increasing pain, volume and oedema formation in the lower leg whilst standing or sitting, during the course of the day or in warm environments, are common in the general population and are indications for treatment with MCS.

Earlier studies reported that venous symptoms, quality of life (QoL) and oedema formation, in patients with lower clinical classes of chronic venous diseases (CVD) (C1s–C3), can be significantly improved by low-pressure compression stockings, compared with placebo stockings.^7,8^ Blättler et al.^9^ reported similar findings for the use of leg compression stockings with an ankle pressure ≤15 mmHg (Table 2). Blazek et al.^10^ reported that, for individuals with professions that require them to stand for prolonged periods, such as hairdressing, and who experience venous symptoms (including leg pain, feelings of swelling and heaviness), wearing leg compression stockings (15–20 mmHg) can alleviate these symptoms. A prospective randomised study that compared thigh-high compression stockings (20–30 mmHg) with sclerotherapy in patients with C1 CVD showed that compression therapy provides significant relief of aching (p < 0.0001), pain (p = 0.002), leg cramps (p = 0.003) and restlessness (p < 0.05), while sclerotherapy provides superior broad-spectrum symptom relief.^11^ Furthermore, in two prospective randomised studies in patients with mild, moderate or severe CVD, Couzan et al.^12,13^ compared progressive compression stockings (i.e. where pressure progressively increases from the ankle to the calf, where the pressure is the highest) with degressive compression stockings (pressure decreases gradually from the ankle to the calf, where the pressure is the lowest). Feelings of heaviness, pain and other lower leg symptoms were significantly alleviated in both groups, but better results were achieved in the progressive compression stockings group.

**Table 2.** Evidence critically reviewed (outcome of the literature search 2007–2015).

Treatment goal	References	Treatment/comparison	Patient information	Outcome
**(a) Chronic venous disorders**				
Improvement of venous symptoms, quality of life and oedema	Blättler et al.^9^	Low-pressure MCS (<10 mmHg) vs. moderate pressure MCS (Ø 15 mmHg) vs. high pressure MCS (Ø 20 mmHg)	Healthy volunteers: n = 40 Dropouts: n = 3	• <10 mmHg MCS: ineffective • 15 mmHg MCS: venous insufficiency symptoms relieved, oedema prevented, MCS well-tolerated • >19 mmHg MCS: not well-tolerated
	Blazek et al.^10^	MCS vs. none	Healthy volunteers (prolonged standing at work): n = 108 Dropouts: n = 10	• MCS: leg pain, feelings of swelling, heaviness and other disturbing feelings alleviated
	Schul et al.^11^	MCS vs. sclerotherapy	Patients: n = 58 Dropouts: n = 9	• MCS: aching, pain, leg cramping and restlessness relieved • Sclerotherapy: additive relief of aching and pain; ongoing symptom relief
	Couzan et al.^12^	MCS degressive vs. MCS progressive in CEAP C0s–C1s	Patients: n = 130 Dropouts: n = 3	• Degressive and progressive MCS: disappearance or major improvement of leg heaviness • Progressive MCS: better tolerated
	Couzan et al.^13^	MCS degressive vs. MCS progressive in CEAP C2s–C5s	Patients: n = 401 Dropouts: n = 20	• Progressive MCS: pain and lower leg symptoms more effectively improved than with degressive MCS • Progressive MCS: easier to apply, no safety concerns
	Mosti et al.^14^	MCS vs. bandage	Patients: n = 30 Dropouts: NA	• MCS: reduced chronic leg oedema nearly as effectively as bandage
	Mosti and Partsch^15^	Bandage followed by MCS vs. MCS followed by second MCS	Patients: n = 28 Dropouts: NA	• Leg volume improvement independent of the pressure applied in first phase (bandages vs. MCS) • Leg volume reduction maintained by superimposing a second MCS
	Sell et al.^16^	MCS vs. surgery	Patient randomized: n = 153 Dropouts: NA	• Surgical elimination of superficial venous reflux: more effective treatment than MCS (assessed by Venous Clinical Severity Score)
	Hagan and Lambert^19^	MCS vs. none	Healthy volunteers: n = 50 Dropouts: n = 3	• MCS: flight-induced ankle oedema, leg pain, discomfort and swelling reduced; energy levels, ability to concentrate, alertness and post-flight sleep improved
	Clarke-Moloney et al.^23^	MCS class 1 (18–21 mmHg) vs. MCS class 2 (23–32 mmHg)	Patients: n = 100 Dropouts: n = 1	• Ulcer recurrence rates: no group difference • Compliance: no group difference • Ulcer recurrence rates: lowest in compliant patients, regardless of compression levels
Improvement of venous leg ulcer healing	Brizzio et al.^17^	MCS vs. bandages	Patients: n = 60 Dropouts: n = 5	• Healing rate and time to healing: no group difference • Pain alleviated equally in both groups
	Finlayson et al.^18^	MCS vs. bandages	Patients: n = 103 Dropouts: n = 16	• Healing of venous leg ulcers equally effective with both systems, but more rapid response with bandages
	Kapp et al.^24^	MCS moderate pressure vs. MCS high pressure	Patients: n = 100 Dropouts: n = 69	• Non-compliant patients: wound recurrence 9 times more likely • Moderate compression: risk recurrence three times greater than with high compression
	Dolibog et al.^27^	IPC vs. MCS vs. bandages	Patients: n = 70 Dropouts: NA	• Wound size reduction and percentage of wounds healed significantly higher in groups receiving IPC or MCS than in groups using short-stretched bandages
	Ashby et al.^28^	MCS vs. bandages	Patients: n = 457 Dropouts: n = 14	• Both treatments equally effective at healing venous leg ulcers • Higher rate of treatment change in MCS group
Reduction of side effects after venous interventions/improvement of therapeutic outcome after venous interventions	Kern et al.^31^	MCS vs. none (after C1 sclerotherapy)	Patients: n = 100 Dropouts: n = 4	• Three weeks of MCS wearing enhanced sclerotherapy efficacy by improving clinical vessel disappearance
	Houtermans-Auckel et al.^37^	MCS vs. none (following 3 days of bandages) (after surgery)	Patients: n = 104 Dropouts: n = 8	• No additional postoperative benefit of MCS longer than 3 days after stripping (with respect to limb oedema, pain, complications and return to work)
	Biswas et al.^38^	1 week TPS vs. 3 weeks’ TPS (after surgery)	Patients: NA	• No benefit from three weeks’ TPS after uncomplicated vein surgery (with respect to postoperative pain, number of complications, time to return to work, patient satisfaction)
	Mariani et al.^39^	MCS vs. bandages	Patients: n = 60 Dropouts: NA	• MCS: less oedema, improved QoL and compliance
	Mosti et al.^40^	MCS vs. bandage vs. eccentric compression pad + MCS (after surgery)	Patients included: n = 54 Dropouts: NA	• Pads + MCS: best results with respect to pain reduction and haematoma • Major adverse events: more frequent in MCS group • Minor adverse events: reported in all groups, most frequent in pad and MCS group
	Benigni et al.^41^	MCS + compression pad vs. MCS alone (after surgery)	Patients: n = 54 Dropouts: n = 1	• Pad addition underneath MCS: postoperative pain significantly reduced
	Lugli et al.^42^	Eccentric compression vs. none (after laser)	Patients: n = 200 Dropouts: NA	• Eccentric compression: postoperative pain intensity greatly reduced
	Bakker et al.^43^	MCS for 48 h vs. MCS for 7 days (after EVL ablation)	Patients: n = 86 Dropouts: n = 10	• Compression for longer than 48 h reduces pain and improves physical function during the first week after treatment
	Reich-Schupke et al.^44^	Low pressure MCS (18–22 mmHg) vs. moderate pressure MCS (23–32 mmHg)	Patients: n = 88 Dropouts: NA	• 23–32 mmHg MCS are superior to 18–21 mmHg MCS in faster resolution of oedema and feelings of pain, tightness, and discomfort of the leg in the first week after varicose vein surgery, but not in the longer post-surgical period up to six weeks
	Hamel-Desnos et al.^45^	MCS vs. none (after sclerotherapy)	Patients: n = 60 Dropouts: NA	• No difference between both groups after sclerotherapy, when comparing efficacy, side effects, satisfaction, symptoms and QoL
**(b) Acute venous disorders**				
Reduction of pain	Kahn et al.^51^	MCS vs. placebo MCS	Patients: n = 806 Dropouts: n = 114 2–3 weeks after DVT	• No difference in pain score between groups • No evidence for subgroup interaction by age, sex or anatomical event of DVT
Reduction of thrombus growth	Boehler et al.^54^	MCS vs. none	Patients: n = 80 Dropouts: n = 7	• MCS: no significant additional benefit to LMWH and NSAIDS in the treatment of SVT after 3 weeks (with respect to pain, NSAID consumption, thrombus length, erythema) • MCS: faster thrombus regression at Day 7
DVT prophylaxis in medical patients and long-distance travellers	Gao et al.^83^	Preoperative TPS + IPC during operation vs. preoperative TPS + none during operation	Patients: n = 108 Dropouts: NA	• TPS + IPC: thrombosis more effectively prevented than with TPS alone (after gynaecological pelvis surgery)
	Cohen et al.^85^	TPS + fondaparinux vs. fondaparinux + none	Patients: n = 856 Completed study: n = 151 Ultrasonographers were blinded to treatment	• TPS: no additional benefit when used with fondaparinux over the use of fondaparinux alone (after hip surgery)
	Camporese et al.^86^	TPS vs. LMWH 7 days vs. LMWH 14 days	Patients: n = 1761 Dropouts: n = 9	• LMWH: reduced a composite end point asymptomatic DVT, symptomatic venous thromboembolism and mortality more than TPS (after knee arthroscopy)
	Chin et al.^87^	TPS vs. intermittent pneumatic compression vs. LMWH vs. none	Patients: n = 440 Dropouts not discussed Ultrasonographers were blinded to treatment	• DVT prevalence significantly lower with IPC or LWMH (but not with TPS) compared to control (after knee arthroscopy)
DVT prophylaxis in stroke patients	Dennis et al.^92^	TPS vs. none	Patients: n = 2518 The study was outcome-blinded	• TPS: risk of DVT development after acute stroke not reduced • CS: skin breaks, ulcer, blisters, skin necrosis more common when compared to patients without TPS
	CLOTS Trial Collaboration^93^	TPS thigh-length vs. knee-length	Patients: after stroke n = 3114 Dropouts at first screening: n = 302 Dropouts at second screening: n = 1832 Ultrasonographers were blind to treatment	• Thigh-length stockings: occurrence of proximal DVT less frequent than with below-knee stockings
Prevention of PTS	Prandoni et al.^61^	MCS thigh-length vs. MCS knee-length	Patients: n = 267 Dropouts: n = 11	• Thigh-length MCS: no better protection against PTS than below-knee MCS + less-well tolerated
	Aschwanden et al.^62^	MCS 26–31 mmHg vs. none	Patients: n = 169 Dropouts: n = 45 The study lacks adequate power to show a significant difference for the primary outcome	• Prolonged compression therapy after proximal DVT: symptoms significantly reduced + post-thrombotic skin changes may be prevented
	Kahn et al.^64^	Active MCS 30–40 mmHg vs. placebo MCS (applied 2–3 weeks after acute DVT)	Patients: n = 806 Dropouts: n = 114 Authors use the term ‘masking’ rather than ‘blinding’ but state that most patients and investigators were unaware of treatment allocation.	• MCS: PTS not prevented after a first proximal DVT
	Jayaraj and Meissner^67^	MCS 30–40 mmHg vs. none	Patients: n = 69 Dropouts at 12 months: n = 14 Dropouts at 24 months: n = 37 Assessor (different from investigator who initially saw patient) was blinded to treatment allocation	• MCS: PTS incidence was lower than in control group at 1- and 3-month cutoff, but not at later visits
Therapy of PTS	Lattimer et al.^73^	MCS 18–21 mmHg below- or above-knee vs. MCS 23–32 mmHg below- or above-knee	Patients: n = 34 (40 legs – 6 had bilateral disease) Each patient acted as their own control and were tested with all four stockings in random order	• Compression: haemodynamic parameters on air plethysmography significantly improved • Haemodynamic benefit not significantly changed with the class or length of stocking
**(c) Lymphatic disorders**				
Prevention of lymphoedema	Stuiver et al.^96^	MCS 23–32 mmHg vs. none	Patients: n = 80 After inguinal lymph node dissection Dropouts at 6 months: n = 11 Dropouts at 12 months: n = 12 The number of dropouts was higher than expected resulting in a larger chance of a type-II error. RR were stable throughout the study	• No significant differences between groups in oedema incidence, median time to oedema occurrence, genital oedema incidence, complication frequency, QoL or body image (after inguinal lymph node dissection)
Improvement of lymphoedema	Dayes et al.^98^	Daily manual lymphatic drainage + bandaging + compression sleeve 30–40 mmHg vs. compression sleeve 30–40 mmHg	Patients: n = 103 Dropouts: n = 8 Assessor blinded to treatment allocation Circumferential tape measurements were used to calculate arm volumes	• No significant improvement in lymphoedema with decongestive therapy compared to compression garments alone

DVT: deep vein thrombosis; IPC: intermittent pneumatic compression; LMWH: low-molecular-weight heparin; MCS: medical compression stockings; NA: not applicable; NSAIDS: non-steroidal anti-inflammatory drugs; PTS: post-thrombotic syndrome; RCT: randomised controlled trials; QoL: quality of life; TPS: thromboprophylactic stockings.

Mosti et al.^14^ have demonstrated that compression stockings exerting a pressure of approximately 30 mmHg are nearly as effective for reducing chronic leg oedema as high-pressure bandages with an initial pressure over 60 mmHg. In another study, Mosti et al.^15^ randomised 40 legs (28 patients) with chronic venous oedema to either short-stretch bandages of initial median pressure 67 mmHg (interquartile range (IQR): 55.7–73.0) applied weekly for two weeks, followed by an elastic stocking for two weeks (group A) or an initial light stocking of median pressure 24.5 mmHg (IQR: 21.2–26.5) for 1 week followed by superimposing a second stocking for three weeks (group B). They reported an initial improvement in leg volume at one week that was independent of the pressure applied, with the reduction maintained by superimposing a second stocking. In a randomised controlled setting, Sell et al.^16^ compared the effectiveness of compression stockings with surgery in patients with uncomplicated varicose veins. At two years, treatment effectiveness (as measured by the venous clinical severity score (VCSS) and the venous segmental disease score (VSDS)), was demonstrated by both treatment modalities, but was greater with surgery. Brizzio et al.^17^ compared the effect of multiple-layer short-stretch bandages with compression stockings (15–25 mmHg) on the rate of healing, pain and QoL, in patients with venous leg ulcer (VLU), and where local pressure over the ulcer was increased by pads. In both groups, pain was reduced by 50%. Healing rates at 90 days were similar (36% and 48% for compression stockings and bandages, respectively) and time to healing was identical. In an RCT comparing the effectiveness of a four-layer compression bandage (CB) system and a Class 3 (30–35 mmHg) compression hosiery system on healing and QoL in patients with VLUs, Finlayson et al.^18^ reported that, at 24 weeks, there was no significant difference between groups in healing, QoL or pain measures, although a four-layer system produced a more rapid response.

Long-distance travellers should benefit from stockings for the prevention of oedema, especially when combined with mobilisation. An RCT conducted in Australia demonstrated that low-ankle pressure graduated compression tights (GCTs) reduce flight-induced ankle oedema and subjectively rated travel symptoms of leg pain, discomfort and swelling, and improve energy levels, ability to concentrate, alertness and post-flight sleep.^19^

For the majority of studies discussed, different compression pressure levels have been compared. It is important to highlight that even low-pressure MCS (10–20 mmHg) are able to reduce symptoms and oedema. In consequence, the pressure level should be adapted to the severity of the disease and limited to the lowest pressure leading to symptom and oedema relief. This will also improve patient compliance. Finally, we conclude that all levels of compression improve venous symptoms and oedema.

Recommendation 1: We recommend the use of MCS to alleviate venous symptoms in patients with CVD (GRADE 1B)

Recommendation 2: We recommend the use of MCS to improve QoL and venous severity scores in patients with CVD (GRADE 1B)

Recommendation 3: We recommend the use of MCS to prevent leg swelling in patients with CVD and in healthy individuals at risk of leg swelling (e.g. during long flights; occupational leg swelling) (GRADE 1B)

Recommendation 4: We recommend the use of MCS to reduce leg swelling in patients with CVD and occupational leg swelling (GRADE 1B)

#### Improvement of skin changes caused by chronic venous disease

The improvement of skin changes – including eczema, induration and lipodermatosclerosis, caused by chronic venous insufficiency (CVI) – by compression therapy is regularly observed in routine clinical practice. However, there is a paucity of evidence from RCTs. The recommendation to use MCS for the improvement of skin changes in general is therefore based on low-level evidence.

Recommendation 5: We suggest MCS for the improvement of skin changes in patients with CVD (GRADE 1C)

Vandongen and Stacey^20^ reported that MCS reduced the area of lipodermatosclerosis in patients with healed venous ulceration. A total of 153 patients with recently healed VLUs were randomised to either below-knee graduated MCS or no stockings. In patients in the compression group, the area of lipodermatosclerosis had reduced significantly, compared with the control group, after six months (p = 0.01) and 12 months (p = 0.04). Within two years of patients entering the study, the area of lipodermatosclerosis was significantly larger in patients with ulcer recurrence than in those who did not re-ulcerate (293 cm^2^ vs. 50 cm^2^). Lipodermatosclerosis was determined both visually and by palpating the leg. A line was drawn on the skin and along the border of the lipodermatosclerosis, traced onto polythene and the area measured using planimetry.

Recommendation 6: We recommend MCS for the improvement of lipodermatosclerosis in patients with CVD (GRADE 1B)

#### Prevention of venous leg ulcer recurrence

Following VLU healing, the ulcer recurrence rate depends on the compensation of the venous insufficiency (either by ablation of superficial venous reflux, improvement of deep venous pathology, where possible, or compression treatment). In a Cochrane review that included RCTs published until June 2000, Nelson et al.^21^ investigated the effect of compression on VLU recurrence. No studies compared compression versus no compression, while one RCT compared ‘high’ compression with ‘moderate’ compression MCS and one study investigated different medium-pressure MCS. Compliance rates were significantly higher with medium compression vs. high compression MCS, and wearing MCS reduced the recurrence rates significantly. Nelson et al.^22^ compared the effectiveness of a ‘moderate’ (18–24 mmHg) and a ‘high’ (25–35 mmHg) compression MCS on venous ulcer recurrence rates in patients with recently healed VLU, with a five-year follow-up. There was no significant difference between the two groups but the recurrence rate in the ‘high’ compression MCS group was lower.

Clarke-Moloney et al.^23^ randomised 100 patients with healed VLU to European Class 1 (18–21 mmHg) or Class 2 (23–32 mmHg) MCS. After 12 months’ follow-up, there was no statistically significant difference in the ulcer recurrence rate between MCS classes, although a greater number of patients in the Class 1 group developed a recurrence and non-compliant patients were at significantly greater risk of recurrence (p ≤ 0.0001). Patients who were compliant with MCS, regardless of the compression level, had the lowest venous ulcer recurrence rates. In a double-blind RCT conducted within a nursing home setting, Kapp et al.^24^ compared the effectiveness of a 23–32 mmHg and a 34–46 mmHg compression stocking on venous ulcer recurrence. The overall adherence to treatment was low (44%). Non-adherence was significantly higher in the high-pressure MCS group (p = 0.003). Risk of recurrence was greater in the ‘moderate’ compression group than the ‘high’ compression group. Adherence to treatment was found to significantly predict ulcer recurrence (p = 0.005).

The results of these studies show a trend of lower rates of venous ulcer recurrence with higher-compression MCS. However, compliance was lower in the ‘high’ compression groups compared with the ‘moderate’ compressions groups. In all studies, compliance with compression treatment significantly reduced VLU recurrence.

Recommendation 7: We recommend the use of MCS to reduce recurrence of VLU (GRADE 1A)

#### Improvement of venous leg ulcer healing

VLUs are one of the most important complications of CVI and they have a great impact on patients’ QoL. CBs are widely used in VLU treatment. A properly applied CB is effective but can be associated with problems of pressure drop during the first hours and a lack of standardisation, especially if the patient applies the bandages themselves. Thus, CBs should be applied by trained staff.

In past decades, special ulcer MCS (‘ulcer kits’) have been developed that comprise a two-layer stocking system. The understocking keeps the ulcer-dressing in place and can be used overnight, whilst an additional higher-pressure overstocking is usually worn during the day.

Following an earlier study by Partsch and Horakova,^25^ who reported that MCS for venous ulcers may be an effective approach for fit and cooperative patients, Jünger et al.^26^ reported that ulcer MCS (ulcer kit), when compared with standard CB, were superior in improving the healing rate of VLUs of a moderate size. Time to healing was similar in both groups. As discussed earlier, similar results were reported by Brizzio et al. and also in comparison with bandages.^17^

In a prospective, clinical pilot study, Dolibog et al.^27^ randomised 70 patients with unilateral VLUs to compression therapy by intermittent pneumatic compression, MCS or short-stretch CB for 15 days. All patients received saline-soaked gauze dressings along with micronised purified flavonoid fraction, diosmin, hesperidin and oral Daflon 500 mg once daily. Wound size reduction and percentage of wounds healed were significantly higher in the groups receiving IPC or stockings than in the groups using short-stretch bandages.

Ashby et al.^28^ randomised 457 patients out of a total of 3411 ulcer patients, presenting with VLUs of median areas 4.1 cm^2^ and 3.7 cm^2^ to either two-layer ulcer MCS (ulcer kit) or four-layer bandage treatment, respectively. Median time to ulcer healing was 99 days (95% confidence interval (CI) 84–126) in the hosiery group and 98 days (95% CI 85–112) in the bandage group. The ulcer healing rate was similar in the two groups (70.9% hosiery and 70.4% bandage). Finlayson et al.^18^ reported results that were similar to these, although in their study the four-layer system showed a more rapid response.

Based on the current literature, the healing rates of VLUs are comparable between ulcer MCS (ulcer kit) and CB systems. This does not apply for circumferential ulcers or ulcers of larger size.

Recommendation 8: We recommend the use of ulcer MCS (‘ulcer kits’) to improve VLU healing (GRADE 1A)

Recommendation 9: We recommend the use of ulcer MCS (‘ulcer kits’) to reduce pain in patients with VLU (GRADE 1A)

#### Prevention of clinical progression in chronic venous disease

Prevention of CVD progression, such as from lower clinical classes to more severe disease, including VLU, is one of the main goals of treatment. There is insufficient information from RCTs on the prevention of CVD progression by MCS that would allow for an evidence-based recommendation. However, in a case–control study by Kostas et al.,^29^ who investigated CVD progression in the contralateral leg of patients treated for varicose veins, non-compliance with compression was reported as one of the risk factors for overall CVD progression.

Recommendation 10: Insufficient data are available on the use of MCS for the prevention of CVD progression, so we recommend further studies are needed to be able to make evidence-based recommendations

#### Reduction of side effects after venous interventions

The use of post-treatment compression to reduce side effects such as pain, oedema, bruising and thromboembolic events is suggested in most guidelines, in regard to venous interventions for varicose veins including high ligation and stripping, endovenous thermal ablation or sclerotherapy. The duration of compression therapy is not clearly defined in most of the guidelines and, over the past decade, endovenous procedures have become significantly less invasive, as a result of the introduction of new devices and methods.

Most RCTs have been limited to great saphenous vein (GSV) interventions. One paper on compression after interventions for subcutaneous varicose veins suggests that less traumatizing surgical methods may not need post-procedural MCS.^30^

Results from one RCT are available after sclerotherapy in C1 varicose veins.^31^ Therefore, evidence-based recommendations can only be given for C1 varicose veins.

Older studies have compared different types or durations of compression therapy without concluding whether compression after interventions is beneficial or not, or on the optimal treatment duration.^32–36^

In a more recent RCT, Houtermans-Auckel et al.^37^ reported that wearing MCS for four weeks after high ligation and stripping of the GSV, following CB use for three days, had no additional benefit on limb oedema, pain, complications and return to work. In a randomised study comparing thromboembolic-deterrent (TED) stockings for one or three weeks after GSV, high ligation and stripping, and three days of postoperative bandaging, Biswas et al.^38^ reported no benefit in wearing TED stockings for more than one week with respect to postoperative pain, number of complications, time to return to work and patient satisfaction for up to 12 weeks following surgery. Mariani et al.^39^ compared MCS (23–32 mmHg) with CB after varicose vein surgery, and reported no difference in postoperative pain, but improvement of oedema and QoL in the stocking group after seven days. A study by Mosti et al.^40^ included 54 patients who had invagination stripping of the GSV and side-branch evulsion under local anaesthesia and were treated for one week, postoperatively, either by thigh-length compression stockings, adhesive bandages or eccentric compression pads fixed with tapes and a superimposed thigh-length stocking on top. Effective reduction of pain and hematoma was obtained with a high local pressure by eccentric compression pads taped to the skin along the stripping channel and a compression stocking on top. Benigni et al.^41^ compared pain intensity on Days 1 and 7 and global mean pain during the week following stripping of the GSV in patients for whom either a pad had been added at thigh level under an MCS or patients with an MCS only. Pain was significantly reduced in the pad plus MCS group (p < 0.0001). Lugli et al.^42^ compared postoperative pain for one week after endovenous laser ablation of the GSV in patients using a special crossed-tape technique that produces higher eccentric compression, compared with those not using pads. In the group using the tape technique, postoperative pain was significantly reduced (p < 0.001).

Bakker et al.^43^ reported that MCS for periods longer than two days after endovenous GSV ablation without simultaneous phlebectomies reduce pain and improve physical function during the first week after treatment. Reich-Schupke et al.^44^ reported that 23–32 mmHg MCS are superior to 18–21 mmHg MCS, providing faster resolution of clinical and ultrasound-verified oedema and feelings of pain, tightness, and discomfort of the leg in the first week after varicose vein surgery, but not in the longer post-surgical period up to six weeks. Regarding pain or complications after foam sclerotherapy of the GSV, Hamel-Desnos et al.^45^ found no significant difference between patients who were wearing 15–20 mmHg thigh-high MCS and those without compression.

Kern et al.^31^ reported that wearing MCS (23–32 mmHg) for three weeks can enhance the efficacy of sclerotherapy of C1 varicose veins by improving clinical vessel disappearance.

Most of the available studies have limitations. In many cases, the interface pressure is not reported or very low. The data, however, show no benefit of MCS for time periods of longer than a week.

Three studies have shown that local high interface pressure, along the GSV at the thigh, can reduce pain.^40–42^

In summary, one week of MCS treatment using thigh-length 20–40 mmHg stockings after GSV interventions can be recommended. Pressure enhancement along the treated vein with additional eccentric padding may be helpful.

Recommendation 11: We recommend the use of MCS in the initial phase after GSV treatment to reduce postoperative side effects (Grade 1B)

Recommendation 12: We recommend additional eccentric compression to enhance the effectiveness of MCS in the reduction of postoperative side effects (Grade 1B)

#### Improvement of therapeutic outcome after venous interventions

The clinical benefit of ongoing MCS treatment after successful correction of pathological changes in the superficial system, or after the reduction of recurrent varicose veins, warrants recommendation of long-term MCS use in these scenarios. Older studies have been unable to show a benefit for intermediate- or long-term use of MCS after venous interventions.^32–36^ A better cosmetic outcome after three weeks of compression stocking was shown in only one trial in which small spider veins had been treated.^31^ The more recent studies either did not follow the patients for a long enough period of time or had failed to demonstrate benefits from ongoing MCS use after the initial postoperative period.^37–42,45^ However, not all patients return to asymptomatic clinical class C0 after venous interventions, even if they have improved clinically, and so may still require MCS treatment. These patients include those who are post-thrombotic after superficial insufficiency treatment, or patients with healed or active VLU, after varicose vein treatment. Prevention of ulcer recurrence still includes MCS use.

Recommendation 13: We do not recommend routine, prolonged use of MCS for improving clinical success after GSV interventions, except for those patients with ongoing symptomatic CVD that benefit from a continued MCS treatment (Grade 1B)

Recommendation 14: We suggest the use of MCS after liquid sclerotherapy of C1 veins to achieve better outcomes (Grade 2B)

## Patients with acute venous disorders

### Deep vein thrombosis

Use of compression in combination with an effective anticoagulant to mobilise patients with acute DVT has long been used in Europe, and is the basis for modern home-based therapy.^46^ Home treatment became accepted for the management of acute DVT after reports that walking with compression does not lead to a higher incidence of pulmonary embolism (PE), compared with bedrest.^47,48^ There is, however, a paucity of evidence from RCTs on the benefits of compression for acute DVT.

### Reduction of pain

In a study in patients with proximal DVT, Blättler and Partsch^49^ assessed the rate of clinical improvement with compression plus immediate deliberate ambulation, in comparison with bedrest without compression, using a visual analogue scale (VAS) to monitor pain and a modified Lowenberg test to monitor provoked pain. The study demonstrated superiority of compression (stockings or bandages), over bedrest without compression, for the reduction of pain.^49^ Roumen-Klappe et al.^50^ evaluated the effect of immediate bandaging vs. no bandaging on the development of PTS. They reported that improvement of clinical symptoms and decrease of leg circumference was significantly better on Day 7 after compression, compared with no compression.

However, it has been reported that when compression is initiated after two weeks or more, there is no difference between compression- and placebo-stockings in the resultant pain levels.^51^

### Reduction of oedema

Swelling of the affected leg – a sign that is easily quantifiable in acute proximal DVT – is rarely reported in studies.

Blättler and Partsch^49^ measured differences in maximal calf circumference in patients with unilateral, acute proximal DVT and reported that, in the first nine days, oedema was significantly reduced with compression, compared with bedrest and no compression (p < 0.001).

### Improvement of walking and QoL

Walking ability, as assessed by quantifying the daily walking distance, was investigated only in one study.^49^ Compression from bandages and stockings was able to increase the mean daily walking distance from 2 km (at Day 1), to 4 km (at Day 9). A significant improvement in QoL parameters – particularly those related to physical functioning – was demonstrated in the group using compression, compared with those using bedrest and no compression (p < 0.05 for stockings; p < 0.001 for bandages).^49^

Recommendation 15: We recommend immediate compression to reduce pain and swelling, thereby allowing instant mobilisation in acute DVT (Grade 1B)

### Reduction of thrombus growth

Arpaia et al.^52^ randomly assigned 73 patients with DVT to elastic compression hosiery starting either immediately after diagnosis or two weeks afterwards. The residual thrombus was measured by compression ultrasonography after 14 and 90 days. The investigators reported that, in the group treated with early compression, there were significantly more recanalised venous segments than in the group treated with delayed compression. Additionally, recanalisation of popliteal DVT veins, expressed as the reduction of vein diameter, was better in the early compression group than in controls at both Day 14 and 90. Blättler and Partsch^49^ reported less thrombus progression with compression, compared with no compression, as assessed by ultrasound.

Recommendation 16: We recommend immediate compression and mobilisation in addition to anticoagulation to avoid thrombus propagation in acute DVT (Grade 1B)

### Superficial vein thrombosis

A Cochrane review of RCTs evaluating topical, medical and surgical treatments for superficial thrombophlebitis included several RCTs that, over the past few years, have reported beneficial effects of different anticoagulation regimens with compression stockings as an adjunct treatment modality in superficial vein thrombosis (SVT).^53^ Boehler et al.^54^ compared the effect of thigh-length compression stockings (21–32 mmHg) versus no compression for a period of three weeks, to understand the therapeutic effect on isolated SVT of the legs. All patients received low-molecular-weight heparin (LMWH), and non-steroidal anti-inflammatory drugs (NSAIDs) were allowed. The primary outcome variable was the reduction of pain, as assessed by a VAS and the Lowenberg test. Secondary outcomes were the consumption of analgesics, thrombus length, skin erythema, D-dimer, and QoL. There was no significant difference between the groups for all tested outcome variables. At Day 7, patients in the compression stocking group had experienced a significantly faster thrombus regression (p = 0.02). The outcome of this study does not exclude potential benefits of stronger compression bandages, worn day and night. Experience from routine practice, as highlighted in the 2012 SVT consensus statement, has emphasized that compression of the thrombosed vein relieves the symptoms of SVT and accelerates healing.^55^

Recommendation 17: We recommend MCS in patients with SVT (Grade 1C).

In patients with SVT who are treated with LMWH, aside from a reduction of thrombus growth after 1 week, an additional benefit for symptomatic outcomes has not been demonstrated

## Post-thrombotic syndrome

Post-thrombotic syndrome (PTS) is a common complication of DVT caused by chronic damage to the venous outflow.^56^ Besides the mechanical flow disturbances due to flow obstruction and reflux, a chronic inflammatory process with ongoing remodelling of the venous wall is also responsible for clinical symptoms, and these have been featured in the scoring system proposed by Villalta and Prandoni.^57^ This score records the presence and severity of five symptoms (cramping, heaviness, pain, paraesthesia, pruritus) and six clinical signs (calf tenderness, induration, oedema, pigmentation, redness, venous ectasia) with the inclusion of venous ulceration as the most severe stage.^3^

The additional use of the CEAP system has been recommended to provide further information.^58^ Importantly, clinical changes should also be described in the acute phase of DVT.

Two RCTs have separately reported the benefits of wearing elastic compression stockings (ECS), in terms of the risk of PTS.^59,60^ In a subsequent study, Prandoni et al.^61^ stated that thigh-high compression stockings were no more effective than knee-high stockings, but were less well tolerated. Aschwanden et al.^62^ conducted an RCT whereby patients were assessed six months after routine compression with MCS; it was reported that prolonged compression therapy after proximal DVT significantly reduces the symptoms associated with PTS, and may prevent post-thrombotic skin changes. Musani et al.^63^ concluded, from a meta-analysis of five RCTs, that wearing MCS after DVT reduces the incidence of a PTS.

The findings of these studies have been challenged by Kahn et al. in a large multicentre RCT that did not find significant differences in the cumulative incidence of PTS defined with Villalta’s scale between patients who were randomized to 30–40 mmHg stockings or to placebo stockings.^64^ This study has, however, been criticised by a number of groups. Poor compliance, differences in patients’ characteristics, the use of different anticoagulants (including new oral anticoagulants), the use of ‘placebo-stockings’ which may have been effective and the delay in the application of MCS have been suggested as possible explanations for the lack of effectiveness of ECS in this trial.^65,66^ However, it must be highlighted that the studies supporting compression to prevent PTS also have limitations (e.g. lower number of included patients). In a recent RCT,^67^ in which both the Villalta-Prandoni Score and Venous Clinical Severity Score instruments were used to monitor PTS, the MCS group had a lower incidence of PTS, compared with the control group, but only at the one-month cut-off. At 6 or 12 months, no difference in the incidence of PTS was detected between the groups. This result highlights the importance of the immediate application of MCS in the acute phase of DVT.^67^

A meta-analysis of eight RCTs concluded that the evidence to date supports the use of compression for reducing the risk of PTS. Nevertheless, the authors stated that the findings need to be interpreted with caution, in view of the heterogeneity in the studies that were included in the meta-analysis.^68^ Individualising management for patients is undergoing investigation and is expected to provide further insight into how to provide patients with optimal management choices.^69^

The consensus panel concluded that current evidence still supports compression therapy for PTS prophylaxis in clinical practice, at least in symptomatic patients.

Recommendation 18: We recommend the use of MCS as early as possible after diagnosis of DVT in order to prevent PTS (Grade 1B)

Comment: Current evidence still supports compression therapy for PTS prophylaxis in clinical practice, at least in symptomatic patients

### Therapy of PTS

Data on the physical management of PTS are sparse;^66,70,71^ the most valuable data are obtainable from ulcer-healing studies, because VLUs are considered the severest form of post-thrombotic syndrome and PTS are the main cause of the ulceration in many cases. A systematic literature review conducted to identify the most superior compression method for promoting ulcer healing and reducing recurrence in patients with lower extremity venous ulcer disease determined that moderate-quality evidence supports the use of compression and low-quality evidence supports the effect of compression on ulcer recurrence.^72^

Recently, Lattimer et al.^73^ have reported on the benefits of compression on haemodynamic parameters in PTS.

Recommendation 19: We recommend the use of MCS for the treatment of symptomatic PTS (Grade 1B)

## Thromboprophylaxis

Two types of compression garments are used for thromboprophylaxis: thromboprophylactic stockings (TPS) and MCS. TPS with a pressure range between 15 and 18 mmHg should be differentiated from MCS that are specifically designed for patients with venous or lymphatic pathology. TPS are able to decrease the venous diameter in the prone position, thereby increasing venous blood flow velocity, which provides the physical conditions needed to avert blood flow stagnation.^74^ TPS are therefore indicated during bedrest, though not for fully mobilised patients.

MCS that are specifically designed for patients with a venous or lymphatic pathology are also preferred for use in individuals when in sitting positions (e.g. using a wheelchair, during long-distance travel).

### Avoid DVT after surgical interventions

In an era of increasingly well-tolerated and effective anti-thrombotic drugs, the role of TPS has become less prominent, although there is a large body of evidence in support of their effectiveness.

Early pioneering work by Turpie et al.,^75^ using radiolabelled fibrinogen scanning, has demonstrated that TPS alone or in combination with IPC is effective in preventing DVT in neurosurgical patients.

Several RCTs have demonstrated a significant reduction in thromboembolic events in neurosurgical patients when stockings were used in combination with anticoagulants, compared with stockings alone.^76,77^ A Cochrane review that compared TPS alone vs. TPS with other DVT prophylactic methods, based on 19 RCTs, concluded that TPS reduced the incidence of DVT after surgery.^78^ However, the scientific evidence is questionable, in view of the dubious combination of physical and pharmacological prophylaxis used.^78^ In patients for whom anticoagulants are contraindicated, physical modalities – including TPS, as well as IPC – are still recommended.^79–81^

An international consensus report recommends the use of TPS in addition to early ambulation and sufficient hydration, for patients undergoing minor surgery with low risk (low level of evidence). Anticoagulant prophylaxis is recommended for moderate- and high-risk patients undergoing surgery.^82^

In high-risk patients undergoing abdominal surgery, a combination of TPS and IPC is more effective than TPS alone.^83^

Well-fitting stockings are recommended in patients with VLUs or venous leg oedema during and after surgery, independent from thromboprophylaxis.^82^

The question of whether use of TPS offers a further significant benefit as an adjunct to pharmacological thromboprophylaxis in surgical inpatients was investigated in a recent systematic review of 27 RCTs. No clear benefit of adding TPS to pharmacological thromboprophylaxis in such patients could be identified, mainly because of the heterogeneity of the results, which precluded a valid summation analysis.^84^

For patients undergoing orthopaedic surgery, the addition of TPS use to an effective anticoagulant has been questioned.^85–87^

Chin et al.^87^ reported that the use of TPS alone is able to reduce DVT in Asian patients who have had a total knee replacement, in comparison to a control group of patients, although IPC and enoxaparin were found to be more effective.

Recommendation 20: We suggest the use of thromboprophylactic stockings as a basic component of mechanical prophylaxis in patients undergoing major surgery (Grade 2C)

Recommendation 21: Mechanical methods of thromboprophylaxis, including thromboprophylactic stockings, should be considered, especially where anticoagulants are contraindicated (Grade 2B)

### DVT prophylaxis in medical patients and long-distance travellers

In recent years, there has been an increase in research on venous thromboembolism (VTE) prevention in high-risk scenarios, particularly in hospitalised medical patients, outpatients with cancer, chronically immobilised patients, long-distance travellers, and in patients with asymptomatic thrombophilia.

Scurr et al.^88^ described asymptomatic calf vein thrombosis after long-haul flights in 10% of passengers without compression stockings, while those who wore MCS did not develop DVT. Clarke et al.^89^ conducted a Cochrane review to assess the effectiveness of MCS for preventing DVT in long-haul travellers and concluded that airline passengers can expect a substantial reduction in the incidence of symptomless DVT and leg oedema if they wear compression stockings.

Several consensus meetings have proposed the use of LMWH and/or MCS in high-risk long-distance travellers.^90^

Mechanical thromboprophylaxis with MCS or IPC was recommended by Kahn et al.^79^ for acutely ill hospitalised patients at increased risk of thrombosis who are bleeding or are at high risk of major bleeding, at least until the bleeding risk is decreased.

Recommendation 22: We suggest the use of MCS during long-distance travelling, to prevent DVT incidence in patients at risk (Grade 2B); in high-risk patients, we suggest a combination of MCS with anticoagulant thromboprophylaxis (Grade 2C)

### DVT prophylaxis in stroke patients

There is a paucity of information on the prevention of VTE among high-risk patients.^91^ The CLOTS trials, which investigated patients who have had a stroke, provide reliable data. In the outcome-blinded RCT of 2518 patients (CLOTS I), where the primary outcome was the occurrence of symptomatic or asymptomatic DVT in the popliteal or femoral veins, thigh-length TPS caused skin problems in 5% of patients and did not reduce the incidence of DVT.^92^ A comparison between thigh-length and knee-length stockings (CLOTS II) revealed that proximal DVT occurred more often in patients with stroke who wore below-knee stockings than in those who wore thigh-length stockings.^93^

A Cochrane review, conducted to determine whether thigh-high or knee-high TPS would be more effective in postoperative surgical patients, reported that there is insufficient high-quality evidence to answer this question.^94^

The CLOTS III trial demonstrated that IPC is an effective method of reducing the risk of DVT and possibly improving survival in a wide variety of patients who are immobile after stroke.^95^

It may be assumed that TPS may help reduce dependent oedema in stroke patients with reduced mobility.

Recommendation 23: We do not recommend below-knee TPS as the sole method for DVT prophylaxis in stroke patients (Grade 2B)

Recommendation 24: If TPS is considered in stroke patients for DVT prophylaxis, we suggest the use of thigh-length TPS over knee-length TPS stockings (Grade 1B)

## Patients with lymphoedema

### Prevention of lymphoedema

There is paucity of data on the use of MCS for the prevention of lymphoedema. A recent RCT that compared thigh-length 21–32 mmHg stockings with usual care, 6 months after inguinal lymph node resection as a result of cancer, reported no significant differences between groups in the incidence of oedema, median time to the occurrence of oedema, incidence of genital oedema, frequency of complications, health-related QoL (HRQoL), or body image.^96^

Comment: More trials are needed to clarify the potential role of compression stockings for preventing lymphoedema after surgery

### Improvement of lymphoedema

Compression is certainly the most important component in the context of ‘decongestive lymphatic therapy’ (DLT) – both for the treatment and for the maintenance phase. However, data endorsing this concept are lacking.^97^ Appropriately fitting compression garments are recommended for long-term maintenance therapy.

A major problem in assessing the efficacy of compression garments in lymphoedema is that the use of stockings in all studies was combined with additional treatment modalities, and primarily manual lymph drainage (MLD). In an RCT in which 95 patients with breast cancer-related arm lymphoedema were randomly assigned to either compression garments (control) or daily manual lymphatic drainage and bandaging followed by compression garments (experimental), Dayes et al.^98^ were unable to demonstrate a significant difference in improvement in lymphoedema between the two groups. The authors indicated that the failure to detect a difference may have been a result of the relatively small size of the trial.

Johannsson et al.^99^ surveyed the current evidence, in regard to the treatment of lymphoedema, and concluded that less emphasis should be placed on MLD and more on early diagnosis, compression, weight control and exercise, when managing patients with cancer-related lymphoedema.

The published data are mainly on volume changes, while symptoms and QoL assessments are inconsistently reported. The traditional concept of starting lymphoedema therapy by compression bandages (‘therapy phase’) followed by stockings (‘maintenance phase’) is supported by the outcome of an RCT which demonstrated that multilayer bandaging as an initial phase of treatment for lymphoedema patients, followed by hosiery, achieves greater and more sustained limb-volume reduction than hosiery alone.^100^

MCS are mainly used to maintain long-term lymphoedema reduction. There is evidence that high-compression stockings (30–40 mmHg) are effective; generally, the highest level of compression that the patient can tolerate (20–60 mmHg) is likely to be the most beneficial.^101^

Recommendation 25: We recommend the use of MCS for lymphoedema maintenance therapy (Grade 1A)

## Discussion

The place of MCS in managing chronic and acute venous and lymphatic disorders is discussed below. Consideration of the effectiveness of different lengths (below-knee, thigh-length, waist-high, etc.), compression pressures, or degrees of stiffness of MCS has not been included because information about the influence of these factors on therapeutic outcomes is either missing or associated recommendations have been inconsistent.

Importantly, we acknowledge that several studies have limitations that led to lower grading of a number of the recommendations. Limitations include low numbers of patients included in the respective studies, as well as instances where MCS was not compared with no compression or placebo. In some studies, the comparator MCS had different pressure levels or a different compression method was applied; thus, a fair comparison of the interventions was not done.

## Chronic venous disorders

In chronic venous disease, MCS are now a main indication for the improvement of venous symptoms, QoL and oedema (Grade 1B). This is particularly true for the treatment of occupational leg oedema and the reduction of venous symptoms/oedema during long-distance travel (Grade 1B).

The clinical observation that stockings reduce the pigmentation and induration of lipodermatosclerosis and venous eczema has not been confirmed in large clinical trials, because of the methodological difficulties of measurement. More studies are needed to confirm their benefit in treating venous skin changes.

The use of MCS on ulcer recurrence prevention is well documented (Grade 1A). However, evidence on their use in treating established ulcers is less clear. Since the 2008 consensus document,^4^ specially designed ulcer MCS (ulcer kits) have been developed. They consist of a two-layer stocking system for uniform graduated compression and ease of application. Their beneficial effect on VLU healing has been reported in several clinical trials.^26,28^

Although it is intuitive that MCS reduce the relentless radial tension in veins during dependency, there is still insufficient information available to recommend their use for the prevention of CVD progression. Further studies are required for an evidence-based recommendation.

As recommended in most of the current recommendations and guidelines, compression has become standard practice after varicose vein surgery to reduce bruising, pigmentation, pain and oedema, and also to improve efficacy.^102^ Now that venous interventions have become minimally invasive, fewer side effects may be expected. Consequently, the need for compression is less clear. Recent studies indicate that in most of the interventions there is still a benefit of MCS during the first post-interventional week.^38,39,44^ These findings relate especially to the reduction of pain, oedema and bruising.

Information on the long-term benefits derived from the use of MCS in the early postoperative phase is lacking in the literature. Interestingly, some benefit after sclerotherapy in C_1_ varicose veins has been reported.^31^

In patients with ongoing CVD symptoms, despite previous interventions, a continuation of compression therapy with MCS is still indicated. Unfortunately, studies that compare MCS with no compression or placebo compression are rare. More commonly, different types of compression have been compared, but with inconsistent results falling short of any recommendation.

## Acute venous disorders

The recommendation to use MCS in the acute stage of DVT to reduce pain and swelling and allow immediate ambulation is based on two studies.^49,50^ Although this regime has been widely accepted,^103,104^ more studies are needed on optimal compression therapy. Research questions that still need to be addressed include: should the length of the stocking be adjusted to the level and extent of the disease?; what compression strength is best, and for how long?; and should the mobility of the patient be taken into consideration? Ideally, such studies should begin in the acute stage, after confirmation of the diagnosis, and should concentrate not only on the morphology of the thrombus, but also on the reduction of leg swelling, pain, and on improving QoL. Similar considerations should be applied to the use of MCS in SVT. However, to date, only one such RCT has been conducted.^54^ Well-conducted studies will be able to determine the extent and benefit of MCS in the long-term prevention of PTS. Implantable electronic devices sewn into the fabric of the stocking should help improve treatment because they have the potential to provide feedback on usage. This approach may be necessary to record patient compliance, because this remains one of the major challenges in the management of venous or lymphatic disorders.

An alternative approach is to provide some type of awareness training to remind the patient regularly about the usefulness of compression therapy. However, though rewarding, such training is very time consuming. The immediate feeling of relief from pain and swelling in the acute stage of venous thrombosis, combined with the assumption that MCS reduce further thromboembolic events, may be the best argument to maximise patient compliance.

Insufficient data are available from prospective RCTs for identifying those patients requiring treatment with MCS, for the purpose of preventing PTS.

Whilst those patients with proximal DVT and a high thrombus burden seem to be the most suitable for treatment, PTS is frequently seen in patients without a clear diagnosis of previous DVT. This suggests that all patients may benefit from MCS after DVT. In agreement with recommendations 3–5 in this document, a high-level recommendation for using MCS in such patients can be justified (Grade 1B).

TPS were recommended for bedridden patients in the 2008 consensus (Grade 1A).^4^ However, their value has been questioned in the light of recent trials.^78,92,93^ This is because prescription of the newer and very effective anti-thrombotic drugs make it difficult to attribute a potentially positive treatment effect to the use of TPS.

This document recommends the use of TPS as a component of mechanical prophylaxis in patients undergoing major surgery (Grade 2C). Correctly measured TPS should be considered in all patients where anticoagulation is contraindicated (Grade 2B). Stocking–skin interface pressures can be measured easily with small portable devices. They are recommended clinically to ensure a good compression profile, especially for legs of non-standard proportions, and are recommended in all research studies.

## Lymphatic disorders

The beneficial effect of MCS in the maintenance of long-term lymphoedema reduction is undisputed and well documented (Grade 1A). However, detailed studies are required to examine their potential role for the prevention of lymphoedema after radiotherapy, cancer and lymph-node dissection. In selecting outcome parameters for such studies, there should be consideration not only of the oedema, but also of the mobility of the patients, their QoL and the ease of applicability of compression garments.

## Other indications

Compression has an anti-inflammatory effect and is often recommended for inflammatory conditions that have an oedema component. These may include cellulitis, some forms of vasculitis, and systemic medical treatments. Compression stockings may help also in the reduction of oedema and nausea during pregnancy. There is a growing demand for their use in sports medicine where they may have an effect on reducing recovery time. In these cases, compression can be used with good clinical success in daily practice but there is a lack of randomised comparative studies that have examined these indications.^105^

## Contraindications

It is best practice not to offer MCS to patients with severe congestive cardiac failure, as there is a risk that they may develop systemic fluid overload. However, reducing moderate oedemas, without shifting larger blood volumes towards the right heart, can be achieved using light MCS.

Another successful indication for light MCS could be the post-reconstructive oedema after successful bypass surgery. The use of light MCS (15–21 mmHg) in these two indications is based on clinical experience only, and deserves further study in the future.

In patients with critical limb ischaemia, with systolic ankle pressure below 70 mmHg or following arterial bypass grafting, compression stockings are contraindicated as there is a risk of ischaemia or local skin necrosis, especially if the limb is elevated.

There are other local conditions in which stockings may cause damage. These include advanced peripheral neuropathy, fragile tissue paper skin over the bony prominences, dermatitis and allergic reactions to the fabric.

For unusual leg sizes, shapes or deformities, a standard ‘off-the-shelf’ stocking is unlikely to fit the patient. In these cases, MCS should be customised to the individual limb measurements.

## Conclusion

This consensus document reports an update of the scientific evidence on the use of MCS in venous and lymphatic disorders. Compared with the ICC consensus document in 2008, several new RCTs have been published showing the improvements that MCS provide in reducing venous symptoms and signs. Although more research is always required, the place of MCS as a treatment is now firmly established for most venous and lymphatic conditions, as well as for venous symptoms in healthy people. The consensus statement was awarded first prize in the oral abstract presentation section at the 30th Annual Congress of the American College of Phlebology 2016, Anaheim, California.

## References

[1] Agus GB Allegra C Antignani PL et al. Guidelines for the diagnosis and therapy of the vein and lymphatic disorders. Int Angiol 2005; 24: 107–168. 15997218

[2] Nicolaides A Kakkos S Eklof B et al. Management of chronic venous disorders of the lower limbs – guidelines according to scientific evidence. Int Angiol 2014; 33: 87–208. 24780922

[3] Wittens C Davies AH Baekgaard N et al. Editor's choice – management of chronic venous disease: clinical practice guidelines of the European Society for Vascular Surgery (ESVS). Eur J Vasc Endovasc Surg 2015; 49: 678–737. 25920631 10.1016/j.ejvs.2015.02.007

[4] Partsch H Flour M Smith PC et al. Indications for compression therapy in venous and lymphatic disease consensus based on experimental data and scientific evidence. Under the auspices of the IUP. Int Angiol 2008; 27: 193–219. 18506124

[5] Eklof B Rutherford RB Bergan JJ et al. Revision of the CEAP classification for chronic venous disorders: consensus statement. J Vasc Surg 2004; 40: 1248–1252. 15622385 10.1016/j.jvs.2004.09.027

[6] Guyatt G Gutterman D Baumann MH et al. Grading strength of recommendations and quality of evidence in clinical guidelines: report from an American College of Chest Physicians task force. Chest 2006; 129: 174–181. 16424429 10.1378/chest.129.1.174

[7] Benigni J Sadoun S Allaert F et al. Comparative study of the effectiveness of class 1 compression stockings on the symptomatology of early chronic venous disease. Phlebologie 2003; 56: 117–125.

[8] Vayssairat M Ziani E Houot B Placebo controlled efficacy of class 1 elastic stockings in chronic venous insufficiency of the lower limbs. J Mal Vasc 2000; 25: 256–262. 11060420

[9] Blättler W Kreis N Lun B et al. Leg symptoms of healthy people and their treatment with compression hosiery. Phlebology 2008; 23: 214–221. 18806203 10.1258/phleb.2008.008014

[10] Blazek C Amsler F Blaettler W et al. Compression hosiery for occupational leg symptoms and leg volume: a randomized crossover trial in a cohort of hairdressers. Phlebology 2013; 28: 239–247. 22451457 10.1258/phleb.2011.011108

[11] Schul MW Eaton T Erdman B Compression versus sclerotherapy for patients with isolated refluxing reticular veins and telangiectasia: a randomized trial comparing quality-of-life outcomes. Phlebology 2011; 26: 148–156. 21422193 10.1258/phleb.2010.009092

[12] Couzan S Assante C Laporte S et al. Booster study: comparative evaluation of a new concept of elastic stockings in mild venous insufficiency. Presse Med 2009; 38: 355–361. 19059750 10.1016/j.lpm.2008.09.019

[13] Couzan S Leizorovicz A Laporte S et al. A randomized double-blind trial of upward progressive versus degressive compressive stockings in patients with moderate to severe chronic venous insufficiency. J Vasc Surg 2012; 56: 1344–1350. 22592040 10.1016/j.jvs.2012.02.060

[14] Mosti G Picerni P Partsch H Compression stockings with moderate pressure are able to reduce chronic leg oedema. Phlebology 2012; 27: 289–296. 22090466 10.1258/phleb.2011.011038

[15] Mosti G Partsch H Bandages or double stockings for the initial therapy of venous oedema? A randomized, controlled pilot study. Eur J Vasc Endovasc Surg 2013; 46: 142–148. 23683393 10.1016/j.ejvs.2013.04.015

[16] Sell H Vikatmaa P Alback A et al. Compression therapy versus surgery in the treatment of patients with varicose veins: a RCT. Eur J Vasc Endovasc Surg 2014; 47: 670–677. 24675145 10.1016/j.ejvs.2014.02.015

[17] Brizzio E Amsler F Lun B et al. Comparison of low-strength compression stockings with bandages for the treatment of recalcitrant venous ulcers. J Vasc Surg 2010; 51: 410–416. 19879713 10.1016/j.jvs.2009.08.048

[18] Finlayson KJ Courtney MD Gibb MA et al. The effectiveness of a four-layer compression bandage system in comparison with Class 3 compression hosiery on healing and quality of life in patients with venous leg ulcers: a randomised controlled trial. Int Wound J 2014; 11: 21–27. 22716129 10.1111/j.1742-481X.2012.01033.x PMC7950767

[19] Hagan MJ Lambert SM A randomised crossover study of low-ankle-pressure graduated-compression tights in reducing flight-induced ankle oedema. Med J Aust 2008; 188: 81–84. 18205579 10.5694/j.1326-5377.2008.tb01527.x

[20] Vandongen YK Stacey MC Graduated compression elastic stockings reduce lipodermatosclerosis and ulcer recurrence. Phlebology 2000; 15: 33–37.

[21] Nelson EA Bell-Syer SE Cullum NA Compression for preventing recurrence of venous ulcers. Cochrane Database Syst Rev 2000; 4: CD002303–CD002303. 10.1002/14651858.CD002303 11034749

[22] Nelson EA Harper DR Prescott RJ et al. Prevention of recurrence of venous ulceration: randomized controlled trial of class 2 and class 3 elastic compression. J Vasc Surg 2006; 44: 803–808. 17012004 10.1016/j.jvs.2006.05.051

[23] Clarke-Moloney M Keane N O'Connor V et al. Randomised controlled trial comparing European standard class 1 to class 2 compression stockings for ulcer recurrence and patient compliance. Int Wound J 2014; 11: 404–408. 23078587 10.1111/j.1742-481X.2012.01108.x PMC7950819

[24] Kapp S Miller C Donohue L The clinical effectiveness of two compression stocking treatments on venous leg ulcer recurrence: a randomized controlled trial. Int J Low Extrem Wounds 2013; 12: 189–198. 24043677 10.1177/1534734613502034

[25] Partsch H Horakova MA Compression stockings in treatment of lower leg venous ulcer. Wien Med Wochenschr 1994; 144: 242–249. 7856197

[26] Jünger M Wollina U Kohnen R et al. Efficacy and tolerability of an ulcer compression stocking for therapy of chronic venous ulcer compared with a below-knee compression bandage: results from a prospective, randomized, multicentre trial. Curr Med Res Opin 2004; 20: 1613–1623. 15462694 10.1185/030079904X4086

[27] Dolibog P Franek A Taradaj J et al. A randomized, controlled clinical pilot study comparing three types of compression therapy to treat venous leg ulcers in patients with superficial and/or segmental deep venous reflux. Ostomy Wound Manage 2013; 59: 22–30. 23934375

[28] Ashby RL Gabe R Ali S et al. Clinical and cost-effectiveness of compression hosiery versus compression bandages in treatment of venous leg ulcers (Venous leg Ulcer Study IV, VenUS IV): a randomised controlled trial. Lancet 2014; 383: 871–879. 24315520 10.1016/S0140-6736(13)62368-5

[29] Kostas TI Ioannou CV Drygiannakis I et al. Chronic venous disease progression and modification of predisposing factors. J Vasc Surg 2010; 51: 900–907. 20347686 10.1016/j.jvs.2009.10.119

[30] Pittaluga P Chastanet S Value of postoperative compression after mini-invasive surgical treatment of varicose veins. J Vasc Surg Venous Lymphat Disord 2013; 1: 385–391. 26992760 10.1016/j.jvsv.2013.01.003

[31] Kern P Ramelet AA Wutschert R et al. Compression after sclerotherapy for telangiectasias and reticular leg veins: a randomized controlled study. J Vasc Surg 2007; 45: 1212–1216. 17467226 10.1016/j.jvs.2007.02.039

[32] Bond R Whyman M Wilkins D et al. A randomised trial of different compression dressings following varicose vein surgery. Phlebology 1999; 14: 9–11.

[33] Raraty M Greaney M Blair SD There is no benefit from 6 weeks’postoperative compression after varicose vein surgery: a prospective randomised trial. Phlebology 1999; 14: 21–25.

[34] Rodrigus I Bleyn J For how long do we have to advise elastic support after varicose vein surgery? A prospective randomized study. Phlebology 1991; 6: 95–98.

[35] Scurr JH Coleridge-Smith P Cutting P Varicose veins: optimum compression following sclerotherapy. Ann R Coll Surg Engl 1985; 67: 109–111. 3883876 PMC2498276

[36] Shouler PJ Runchman PC Varicose veins: optimum compression after surgery and sclerotherapy. Ann R Coll Surg Engl 1989; 71: 402–404. 2690721 PMC2499037

[37] Houtermans-Auckel J van Rossum J Teijink J et al. To wear or not to wear compression stockings after varicose vein stripping: a randomised controlled trial. Eur J Vasc Endovasc Surg 2009; 38: 387–391. 19608438 10.1016/j.ejvs.2009.05.025

[38] Biswas S Clark A Shields DA Randomised clinical trial of the duration of compression therapy after varicose vein surgery. Eur J Vasc Endovasc Surg 2007; 33: 631–637. 17276100 10.1016/j.ejvs.2006.12.003

[39] Mariani F Marone EM Gasbarro V et al. Multicenter randomized trial comparing compression with elastic stocking versus bandage after surgery for varicose veins. J Vasc Surg 2011; 53: 115–122. 21050700 10.1016/j.jvs.2010.08.033

[40] Mosti G Mattaliano V Arleo S et al. Thigh compression after great saphenous surgery is more effective with high pressure. Int Angiol 2009; 28: 274–280. 19648870

[41] Benigni JP Allaert FA Desoutter P et al. The efficiency of pain control using a thigh pad under the elastic stocking in patients following venous stripping: results of a case-control study. Perspect Vasc Surg Endovasc Ther 2011; 23: 238–243. 22237977 10.1177/1531003511431737

[42] Lugli M Cogo A Guerzoni S et al. Effects of eccentric compression by a crossed-tape technique after endovenous laser ablation of the great saphenous vein: a randomized study. Phlebology 2009; 24: 151–156. 19620697 10.1258/phleb.2008.008045

[43] Bakker NA Schieven LW Bruins RM et al. Compression stockings after endovenous laser ablation of the great saphenous vein: a prospective randomized controlled trial. Eur J Vasc Endovasc Surg 2013; 46: 588–592. 24012465 10.1016/j.ejvs.2013.08.001

[44] Reich-Schupke S Feldhaus F Altmeyer P et al. Efficacy and comfort of medical compression stockings with low and moderate pressure six weeks after vein surgery. Phlebology 2014; 29: 358–366. 23563646 10.1177/0268355513484142

[45] Hamel-Desnos CM Guias BJ Desnos PR et al. Foam sclerotherapy of the saphenous veins: randomised controlled trial with or without compression. Eur J Vasc Endovasc Surg 2010; 39: 500–507. 20097585 10.1016/j.ejvs.2009.11.027

[46] Galanaud JP Laroche JP Righini M The history and historical treatments of deep vein thrombosis. J Thromb Haemost 2013; 11: 402–411. 23297815 10.1111/jth.12127

[47] Aschwanden M Labs KH Engel H et al. Acute deep vein thrombosis: early mobilization does not increase the frequency of pulmonary embolism. Thromb Haemost 2001; 85: 42–46. 11204585

[48] Schellong SM Schwarz T Kropp J et al. Bed rest in deep vein thrombosis and the incidence of scintigraphic pulmonary embolism. Thromb Haemost 1999; 82(Suppl 1): 127–129. 10695503

[49] Blättler W Partsch H Leg compression and ambulation is better than bed rest for the treatment of acute deep venous thrombosis. Int Angiol 2003; 22: 393–400. 15153824

[50] Roumen-Klappe EM den Heijer M van Rossum J et al. Multilayer compression bandaging in the acute phase of deep-vein thrombosis has no effect on the development of the post-thrombotic syndrome. J Thromb Thrombolysis 2009; 27: 400–405. 18480967 10.1007/s11239-008-0229-7

[51] Kahn SR Shapiro S Ducruet T et al. Graduated compression stockings to treat acute leg pain associated with proximal DVT. A randomised controlled trial. Thromb Haemost 2014; 112: 1137–1141. 25183442 10.1160/TH14-05-0430

[52] Arpaia G Cimminiello C Mastrogiacomo O et al. Efficacy of elastic compression stockings used early or after resolution of the edema on recanalization after deep venous thrombosis: the COM.PRE Trial. Blood Coagul Fibrinolysis 2007; 18: 131–137. 17287629 10.1097/MBC.0b013e328011f2dd

[53] Di Nisio M Wichers IM Middeldorp S Treatment for superficial thrombophlebitis of the leg. Cochrane Database Syst Rev 2013; 4: CD004982–CD004982. 10.1002/14651858.CD004982.pub3 17443561

[54] Boehler K Kittler H Stolkovich S et al. Therapeutic effect of compression stockings versus no compression on isolated superficial vein thrombosis of the legs: a randomized clinical trial. Eur J Vasc Endovasc Surg 2014; 48: 465–471. 25116277 10.1016/j.ejvs.2014.06.047

[55] Kalodiki E Stvrtinova V Allegra C et al. Superficial vein thrombosis: a consensus statement. Int Angiol 2012; 31: 203–216. 22634973

[56] Kahn SR The post-thrombotic syndrome: progress and pitfalls. Br J Haematol 2006; 134: 357–365. 16822286 10.1111/j.1365-2141.2006.06200.x

[57] Kahn SR Measurement properties of the Villalta scale to define and classify the severity of the post-thrombotic syndrome. J Thromb Haemost 2009; 7: 884–888. 19320818 10.1111/j.1538-7836.2009.03339.x

[58] Kahn SR Partsch H Vedantham S et al. Definition of post-thrombotic syndrome of the leg for use in clinical investigations: a recommendation for standardization. J Thromb Haemost 2009; 7: 879–883. 19175497 10.1111/j.1538-7836.2009.03294.x

[59] Brandjes DP Buller HR Heijboer H et al. Randomised trial of effect of compression stockings in patients with symptomatic proximal-vein thrombosis. Lancet 1997; 349: 759–762. 9074574 10.1016/S0140-6736(96)12215-7

[60] Prandoni P Lensing AW Prins MH et al. Below-knee elastic compression stockings to prevent the post-thrombotic syndrome: a randomized, controlled trial. Ann Intern Med 2004; 141: 249–256. 15313740 10.7326/0003-4819-141-4-200408170-00004

[61] Prandoni P Noventa F Quintavalla R et al. Thigh-length versus below-knee compression elastic stockings for prevention of the postthrombotic syndrome in patients with proximal-venous thrombosis: a randomized trial. Blood 2012; 119: 1561–1565. 22180438 10.1182/blood-2011-11-391961

[62] Aschwanden M Jeanneret C Koller MT et al. Effect of prolonged treatment with compression stockings to prevent post-thrombotic sequelae: a randomized controlled trial. J Vasc Surg 2008; 47: 1015–1021. 18372153 10.1016/j.jvs.2008.01.008

[63] Musani MH Matta F Yaekoub AY et al. Venous compression for prevention of postthrombotic syndrome: a meta-analysis. Am J Med 2010; 123: 735–740. 20670728 10.1016/j.amjmed.2010.01.027

[64] Kahn SR Shapiro S Wells PS et al. Compression stockings to prevent post-thrombotic syndrome: a randomised placebo-controlled trial. Lancet 2014; 383: 880–888. 24315521 10.1016/S0140-6736(13)61902-9

[65] Labropoulos N Gasparis AP Caprini JA et al. Compression stockings to prevent post-thrombotic syndrome. Lancet 2014; 384: 129–130. 10.1016/S0140-6736(14)61159-4 25016992

[66] Ten Cate-Hoek AJ Elastic compression stockings – is there any benefit? Lancet 2014; 383: 851–853. 24315518 10.1016/S0140-6736(13)62347-8

[67] Jayaraj A Meissner M Impact of graduated compression stockings on the prevention of post-thrombotic syndrome – results of a randomized controlled trial. Phlebology 2015; 30: 541–548. 25059736 10.1177/0268355514544781

[68] Tie HT Luo MZ Luo MJ et al. Compression therapy in the prevention of postthrombotic syndrome: a systematic review and meta-analysis. Medicine (Baltimore) 2015; 94: e1318–e1318. 26252318 10.1097/MD.0000000000001318 PMC4616586

[69] Ten Cate-Hoek AJ Bouman AC Joore MA et al. The IDEAL DVT study, individualised duration elastic compression therapy against long-term duration of therapy for the prevention of post-thrombotic syndrome: protocol of a randomised controlled trial. BMJ Open 2014; 4: e005265–e005265. 10.1136/bmjopen-2014-005265 PMC4158195 25190617

[70] Kolbach DN Sandbrink MW Neumann HA et al. Compression therapy for treating stage I and II (Widmer) post-thrombotic syndrome. Cochrane Database Syst Rev 2003; 4: CD004177–CD004177. 10.1002/14651858.CD004177 14584008

[71] Cohen JM Akl EA Kahn SR Pharmacologic and compression therapies for postthrombotic syndrome: a systematic review of randomized controlled trials. Chest 2012; 141: 308–320. 22315114 10.1378/chest.11-1175

[72] Mauck KF Asi N Elraiyah TA et al. Comparative systematic review and meta-analysis of compression modalities for the promotion of venous ulcer healing and reducing ulcer recurrence. J Vasc Surg 2014; 60: 71S–90S. 24877851 10.1016/j.jvs.2014.04.060

[73] Lattimer CR Azzam M Kalodiki E et al. Compression stockings significantly improve hemodynamic performance in post-thrombotic syndrome irrespective of class or length. J Vasc Surg 2013; 58: 158–165. 23414697 10.1016/j.jvs.2013.01.003

[74] Downie SP Raynor SM Firmin DN et al. Effects of elastic compression stockings on wall shear stress in deep and superficial veins of the calf. Am J Physiol Heart Circ Physiol 2008; 294: H2112–H2120. 18326802 10.1152/ajpheart.01302.2007

[75] Turpie AG Hirsh J Gent M et al. Prevention of deep vein thrombosis in potential neurosurgical patients. A randomized trial comparing graduated compression stockings alone or graduated compression stockings plus intermittent pneumatic compression with control. Arch Intern Med 1989; 149: 679–681. 2645846

[76] Agnelli G Piovella F Buoncristiani P et al. Enoxaparin plus compression stockings compared with compression stockings alone in the prevention of venous thromboembolism after elective neurosurgery. N Engl J Med 1998; 339: 80–85. 9654538 10.1056/NEJM199807093390204

[77] Nurmohamed MT van Riel AM Henkens CM et al. Low molecular weight heparin and compression stockings in the prevention of venous thromboembolism in neurosurgery. Thromb Haemost 1996; 75: 233–238. 8815566

[78] Sachdeva A Dalton M Amaragiri SV et al. Graduated compression stockings for prevention of deep vein thrombosis. Cochrane Database Syst Rev 2014; 12: CD001484–CD001484. 10.1002/14651858.CD001484.pub3 25517473

[79] Kahn SR Lim W Dunn AS et al. Prevention of VTE in nonsurgical patients: antithrombotic therapy and prevention of thrombosis. 9th ed: American College of Chest Physicians Evidence-Based Clinical Practice Guidelines. Chest 2012; 141: e195S–226S. 22315261 10.1378/chest.11-2296 PMC3278052

[80] Falck-Ytter Y Francis CW Johanson NA et al. Prevention of VTE in orthopedic surgery patients: Antithrombotic Therapy and Prevention of Thrombosis. 9th ed: American College of Chest Physicians Evidence-Based Clinical Practice Guidelines. Chest 2012; 141: e278S–325S. 22315265 10.1378/chest.11-2404 PMC3278063

[81] Eppsteiner RW Shin JJ Johnson J et al. Mechanical compression versus subcutaneous heparin therapy in postoperative and posttrauma patients: a systematic review and meta-analysis. World J Surg 2010; 34: 10–19. 20020289 10.1007/s00268-009-0284-z

[82] Nicolaides AN Fareed J Kakkar AK et al. Prevention and treatment of venous thromboembolism – International Consensus Statement. Int Angiol 2013; 32: 111–260. 24402349

[83] Gao J Zhang ZY Li Z et al. Two mechanical methods for thromboembolism prophylaxis after gynaecological pelvic surgery: a prospective, randomised study. Chin Med J (Engl) 2012; 125: 4259–4263. 23217397

[84] Mandavia R Shalhoub J Head K et al. The additional benefit of graduated compression stockings to pharmacologic thromboprophylaxis in the prevention of venous thromboembolism in surgical inpatients. J Vasc Surg Venous Lymphat Disord 2015; 3: 447–455. 26992625 10.1016/j.jvsv.2014.10.002

[85] Cohen AT Skinner JA Warwick D et al. The use of graduated compression stockings in association with fondaparinux in surgery of the hip. A multicentre, multinational, randomised, open-label, parallel-group comparative study. J Bone Joint Surg Br 2007; 89: 887–892. 17673580 10.1302/0301-620X.89B7.18556

[86] Camporese G Bernardi E Prandoni P et al. Low-molecular-weight heparin versus compression stockings for thromboprophylaxis after knee arthroscopy: a randomized trial. Ann Intern Med 2008; 149: 73–82. 18626046 10.7326/0003-4819-149-2-200807150-00003

[87] Chin PL Amin MS Yang KY et al. Thromboembolic prophylaxis for total knee arthroplasty in Asian patients: a randomised controlled trial. J Orthop Surg (Hong Kong) 2009; 17: 1–5. 19398783 10.1177/230949900901700101

[88] Scurr JH Machin SJ Bailey-King S et al. Frequency and prevention of symptomless deep-vein thrombosis in long-haul flights: a randomised trial. Lancet 2001; 357: 1485–1489. 11377600 10.1016/S0140-6736(00)04645-6

[89] Clarke M Hopewell S Juszczak E et al. Compression stockings for preventing deep vein thrombosis in airline passengers. Cochrane Database Syst Rev 2006; 2: CD004002–CD004002. 10.1002/14651858.CD004002.pub2 16625594

[90] Schobersberger W Toff WD Eklof B et al. Traveller's thrombosis: international consensus statement. Vasa 2008; 37: 311–317. 19003740 10.1024/0301-1526.37.4.311

[91] Roderick P Ferris G Wilson K et al. Towards evidence-based guidelines for the prevention of venous thromboembolism: systematic reviews of mechanical methods, oral anticoagulation, dextran and regional anaesthesia as thromboprophylaxis. Health Technol Assess 2005; 9: iii–iv, ix–x, 1–78–iii–iv, ix–x, 1–78. 10.3310/hta9490 16336844

[92] Dennis M Sandercock PA et al. Effectiveness of thigh-length graduated compression stockings to reduce the risk of deep vein thrombosis after stroke (CLOTS trial 1): a multicentre, randomised controlled trial. Lancet 2009; 373: 1958–1965. 19477503 10.1016/S0140-6736(09)60941-7 PMC2692021

[93] CLOTS Trial Collaboration Thigh-length versus below-knee stockings for deep venous thrombosis prophylaxis after stroke: a randomized trial. Ann Intern Med 2010; 153: 553–562. 20855784 10.7326/0003-4819-153-9-201011020-00280

[94] Sajid MS Desai M Morris RW et al. Knee length versus thigh length graduated compression stockings for prevention of deep vein thrombosis in postoperative surgical patients. Cochrane Database Syst Rev 2012; 5: CD007162–CD007162. 10.1002/14651858.CD007162.pub2 PMC11694892 22592717

[95] Dennis M Sandercock P et al. Effectiveness of intermittent pneumatic compression in reduction of risk of deep vein thrombosis in patients who have had a stroke (CLOTS 3): a multicentre randomised controlled trial. Lancet 2013; 382: 516–524. 23727163 10.1016/S0140-6736(13)61050-8

[96] Stuiver MM de Rooij JD Lucas C et al. No evidence of benefit from class-II compression stockings in the prevention of lower-limb lymphedema after inguinal lymph node dissection: results of a randomized controlled trial. Lymphology 2013; 46: 120–131. 24645535

[97] Lee BB Andrade M Antignani PL et al. Diagnosis and treatment of primary lymphedema. Consensus document of the International Union of Phlebology (IUP)-2013. Int Angiol 2013; 32: 541–574. 24212289

[98] Dayes IS Whelan TJ Julian JA et al. Randomized trial of decongestive lymphatic therapy for the treatment of lymphedema in women with breast cancer. J Clin Oncol 2013; 31: 3758–3763. 24043733 10.1200/JCO.2012.45.7192

[99] Johansson K Karlsson K Nikolaidis P Evidence-based or traditional treatment of cancer-related lymphedema. Lymphology 2015; 48: 24–27. 26333211

[100] Badger CM Peacock JL Mortimer PS A randomized, controlled, parallel-group clinical trial comparing multilayer bandaging followed by hosiery versus hosiery alone in the treatment of patients with lymphedema of the limb. Cancer 2000; 88: 2832–2837. 10870068

[101] International Society of Lymphology. The diagnosis and treatment of peripheral lymphedema: 2013 consensus document of the International Society of Lymphology. Lymphology 2013; 46: 1–11. 23930436

[102] Mosti G De Maeseneer M Cavezzi A et al. Society for Vascular Surgery and American Venous Forum Guidelines on the management of venous leg ulcers: the point of view of the International Union of Phlebology. Int Angiol 2015; 34: 202–218. 25896614

[103] Blättler W Gerlach HE Implementation of outpatient treatment of deep-vein thrombosis in private practices in Germany. Eur J Vasc Endovasc Surg 2005; 30: 319–324. 15949958 10.1016/j.ejvs.2005.05.001

[104] Hach-Wunderle V Bauersachs R Gerlach HE et al. Post-thrombotic syndrome 3 years after deep venous thrombosis in the Thrombosis and Pulmonary Embolism in Out-Patients (TULIPA) PLUS Registry. J Vasc Surg Venous Lymphat Disord 2013; 1: 5–12. 26993886 10.1016/j.jvsv.2012.07.003

[105] Veins and Lymphatics. Cinderella indications for compression. *International Compression Club (ICC) Meeting* 5; No 1.

